# Bis[4,5-dimethyl-2-(2-pyrid­yl)-1*H*-imidazole-κ^2^ *N* ^2^,*N* ^3^](1*H*-imidazole-κ*N* ^3^)copper(II) bis­(perchlorate)

**DOI:** 10.1107/S1600536808017273

**Published:** 2008-06-13

**Authors:** Chunyi Liu, Anyu Zhou, Songpei Wang, Zhengping Chen

**Affiliations:** a The Key Laboratory of Nuclear Medicine, Ministry of Health P. R. China, Jiangsu Institute of Nuclear Medicine, Wuxi 214063, People’s Republic of China; b Department of Chemistry & Chemical Engineering, Southeast University, Nanjing 210096, People’s Republic of China

## Abstract

In the title complex, [Cu(C_3_H_4_N_2_)(C_10_H_11_N_3_)_2_](ClO_4_)_2_, the Cu^II^ cation has a distorted trigonal-bipyramidal geometry defined by a CuN_2_N′_2_N′′ donor set. The imidazole ligand is disordered over two orientations of equal occupancy. Two of the perchlorate ion sites are located on a twofold rotation axis, and one of is disordered over two sites of equal occupancy. In the crystal structure there is a two-dimensional infinite network of hydrogen-bonded mol­ecules parallel to the *ab* plane.

## Related literature

For related literature, see: Holm *et al.* (1996 ▶); Huang *et al.* (2004 ▶); Huang *et al.* (2005 ▶); Kapinos *et al.* (1998 ▶); Matthews *et al.* (1998 ▶); Tan *et al.* (1997 ▶).
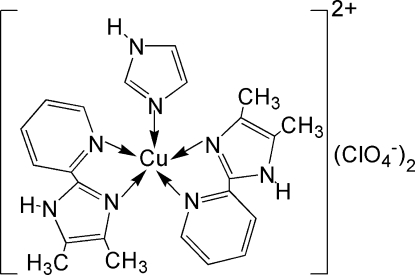


## Experimental

### 

#### Crystal data

[Cu(C_3_H_4_N_2_)(C_10_H_11_N_3_)_2_](ClO_4_)_2_
*M* *_r_* = 676.96
Tetragonal, 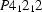

*a* = 14.6374 (5) Å
*c* = 27.3945 (14) Å
*V* = 5869.4 (4) Å^3^
*Z* = 8
Mo *K*α radiation
μ = 0.99 mm^−1^
*T* = 293 (2) K
0.32 × 0.26 × 0.24 mm

#### Data collection

Bruker SMART APEX CCD area-detector diffractometer
Absorption correction: multi-scan (*SADABS*; Bruker, 2000 ▶) *T*_min_ = 0.74, *T*_max_ = 0.79
32170 measured reflections
5775 independent reflections
5315 reflections with *I* > 2σ(*I*)
*R*_int_ = 0.048

#### Refinement

*R*[*F* ^2^ > 2σ(*F* ^2^)] = 0.048
*wR*(*F* ^2^) = 0.117
*S* = 1.08
5775 reflections
410 parameters
H-atom parameters constrained
Δρ_max_ = 0.54 e Å^−3^
Δρ_min_ = −0.64 e Å^−3^
Absolute structure: Flack (1983 ▶), 2433 Friedel pairs
Flack parameter: 0.013 (17)

### 

Data collection: *SMART* (Bruker, 2000 ▶); cell refinement: *SAINT* (Bruker, 2000 ▶); data reduction: *SAINT*; program(s) used to solve structure: *SHELXTL* (Sheldrick, 2008 ▶); program(s) used to refine structure: *SHELXTL*; molecular graphics: *SHELXTL*; software used to prepare material for publication: *SHELXTL*.

## Supplementary Material

Crystal structure: contains datablocks I, global. DOI: 10.1107/S1600536808017273/kj2084sup1.cif

Structure factors: contains datablocks I. DOI: 10.1107/S1600536808017273/kj2084Isup2.hkl

Additional supplementary materials:  crystallographic information; 3D view; checkCIF report

## Figures and Tables

**Table:** 

Cu1—N5	1.977 (3)
Cu1—N2	1.990 (3)
Cu1—N7	2.007 (3)
Cu1—N1	2.129 (3)
Cu1—N4	2.137 (3)

**Table:** 

N5—Cu1—N2	170.20 (15)
N5—Cu1—N7	94.06 (14)
N2—Cu1—N7	95.74 (15)
N5—Cu1—N1	94.06 (13)
N2—Cu1—N1	80.10 (13)
N7—Cu1—N1	127.46 (14)
N5—Cu1—N4	79.52 (13)
N2—Cu1—N4	94.82 (13)
N7—Cu1—N4	123.73 (14)
N1—Cu1—N4	108.79 (13)

**Table 2:** Hydrogen-bond geometry (Å, °)

*D*—H⋯*A*	*D*—H	H⋯*A*	*D*⋯*A*	*D*—H⋯*A*
N3—H3*A*⋯O12	0.86	2.16	3.001 (8)	166
N3—H3*A*⋯O14^i^	0.86	2.15	2.980 (10)	162
N6—H6⋯O21^i^	0.86	2.15	3.009 (5)	175

Symmetry code: (i) 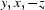.

**Fig. 1.**
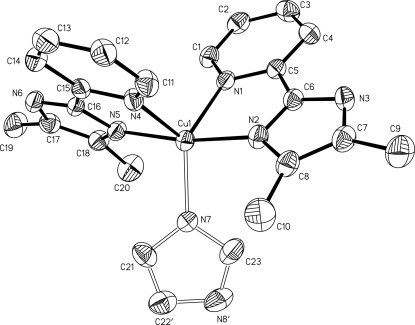
The molecular structure of the complex in (I), showing the atomic labelling. Displacement ellipsoids are shown at the 50% probability level. H atoms, counter ions and one disorder component have been omitted for clarity.

## supplementary crystallographic information

### Comment

Imidazole is ubiquitous in biology and chemistry. It is therefore of interest to synthesize ligands containing imidazole and related heterocyclic system (Tan *et al.*, 1997; Kapinos *et al.*, 1998; Matthews *et al.*, 1998). Imidazole and its derivatives are an important class of heterocycle with N-donor atoms, therefore the investigation of mixed-ligand complexes of a variety of transitional metal ions with imidazole and its derivatives has attracted considerable interest in recent years. The copper-imidazole systems have demonstrated capacities for construction of inorganic-organic hybrid supramolecular isomers (Huang *et al.*, 2004; Huang *et al.*, 2005), and also have profound effects on functions in biological systems (Holm *et al.*, 1996). We report here the crystal structure of the title compound, a mixed-ligand Cu^II^ complex.

The crystal structure of the title compound contains distorted Cu^II^ complexes in which individual Cu centres exist in a CuN_2_N'_2_N'' donor set that defines a distorted trigonal bipyramid geometry. The two N atoms of pyridyl rings and the N atom of imidazole coordinate in a plane around the Cu atom. The N atoms of the two imidazole rings distribute in the axial positions; the Cu1—N2 distance is 1.990 (3) Å. The imidazole ligand is not a ordered system, and the imidazole ring is disordered over two orientations. Two of the perchlorate ion sites (containing Cl1 and Cl2) are located on a twofold rotation axis, and one of these (containing Cl1) displays disorder.

The N3 atom of 4,5-dimethyl-2-(2-pyridyl)imidazole form two hydrogen bonds with the O atom of perchlorate. The N6 atom and N8 atom also each form a hydrogen bond with the O atom of perchlorate; details are presented in Table 2. A two-dimensional infinite network of hydrogen-bonded molecules is present in the structure, running parallel to the *ab*-plane.

### Experimental

The title complex was synthesized by the reaction of 4,5-dimethyl-2- (2-pyridyl)imidazole (0.52 g, 3.0 mmol) and imidazole (0.10 g, 1.5 mmol) with copper(II) perchlorate (0.50 g, 1.5 mmol) dissolved in the methanol (20 ml). Single crystals of (I) suitable for X-ray diffraction were obtained by evaporation of the methanol solution at room temperature.

### Refinement

All H atoms were allowed to ride on their parent atoms at distances of 0.96Å (methyl H), 0.93Å (pyridyl H), 0.93Å (imidazole H) and 0.86Å (N—H imidazole), and with *U*_iso_(H) values of 1.2–1.5 times *U*_eq_ of the parent atom.

### Crystal data

**Table:**

[Cu(C_3_H_4_N_2_)(C_10_H_11_N_3_)_2_](ClO_4_)_2_	*Z* = 8
*M**_r_* = 676.96	*F*_000_ = 2776
Tetragonal, *P*4_1_2_1_2	*D*_x_ = 1.532 Mg m^−^^3^
Hall symbol: P 4abw 2nw	Mo *K*α radiation λ = 0.71073 Å
*a* = 14.6374 (5) Å	Cell parameters from 8320 reflections
*b* = 14.6374 (5) Å	θ = 2.5–23.8º
*c* = 27.3945 (14) Å	µ = 0.99 mm^−^^1^
α = 90º	*T* = 293 (2) K
β = 90º	Bipyramid, green
γ = 90º	0.32 × 0.26 × 0.24 mm
*V* = 5869.4 (4) Å^3^	

### Data collection

**Table:**

Bruker SMART APEX CCD area-detector diffractometer	5775 independent reflections
Radiation source: sealed tube	5315 reflections with *I* > 2σ(*I*)
Monochromator: graphite	*R*_int_ = 0.048
*T* = 273(2) K	θ_max_ = 26.0º
φ and ω scans	θ_min_ = 1.6º
Absorption correction: multi-scan(SADABS; Bruker, 2000)	*h* = −18→14
*T*_min_ = 0.74, *T*_max_ = 0.79	*k* = −18→18
32170 measured reflections	*l* = −33→27

### Refinement

**Table:**

Refinement on *F*^2^	Hydrogen site location: inferred from neighbouring sites
Least-squares matrix: full	H-atom parameters constrained
*R*[*F*^2^ > 2σ(*F*^2^)] = 0.048	*w* = 1/[σ^2^(*F*_o_^2^) + (0.049*P*)^2^ + 5.1204*P*] where *P* = (*F*_o_^2^ + 2*F*_c_^2^)/3
*wR*(*F*^2^) = 0.117	(Δ/σ)_max_ < 0.001
*S* = 1.08	Δρ_max_ = 0.54 e Å^−^^3^
5775 reflections	Δρ_min_ = −0.63 e Å^−^^3^
410 parameters	Extinction correction: none
Primary atom site location: structure-invariant direct methods	Absolute structure: Flack (1983), 2433 Friedel pairs
Secondary atom site location: difference Fourier map	Flack parameter: 0.013 (17)

### Special details

**Table:**

Geometry. All e.s.d.'s (except the e.s.d. in the dihedral angle between two l.s. planes) are estimated using the full covariance matrix. The cell e.s.d.'s are taken into account individually in the estimation of e.s.d.'s in distances, angles and torsion angles; correlations between e.s.d.'s in cell parameters are only used when they are defined by crystal symmetry. An approximate (isotropic) treatment of cell e.s.d.'s is used for estimating e.s.d.'s involving l.s. planes.
Refinement. Refinement of *F*^2^ against ALL reflections. The weighted *R*-factor *wR* and goodness of fit *S* are based on *F*^2^, conventional *R*-factors *R* are based on *F*, with *F* set to zero for negative *F*^2^. The threshold expression of *F*^2^ > σ(*F*^2^) is used only for calculating *R*-factors(gt) *etc*. and is not relevant to the choice of reflections for refinement. *R*-factors based on *F*^2^ are statistically about twice as large as those based on *F*, and *R*- factors based on ALL data will be even larger.

### Fractional atomic coordinates and isotropic or equivalent isotropic displacement parameters (Å^2^)

**Table:**

	*x*	*y*	*z*	*U*_iso_*/*U*_eq_	Occ. (<1)
Cu1	0.49074 (3)	0.81891 (3)	0.002420 (17)	0.04289 (13)	
C1	0.5381 (3)	0.7035 (3)	−0.09104 (15)	0.0491 (10)	
H1A	0.5698	0.7544	−0.1023	0.059*	
C2	0.5351 (3)	0.6266 (3)	−0.12011 (16)	0.0560 (11)	
H2A	0.5649	0.6258	−0.1501	0.067*	
C3	0.4875 (3)	0.5508 (3)	−0.10421 (17)	0.0590 (12)	
H3B	0.4848	0.4985	−0.1234	0.071*	
C4	0.4438 (3)	0.5534 (3)	−0.05947 (17)	0.0547 (10)	
H4A	0.4104	0.5037	−0.0482	0.066*	
C5	0.4516 (3)	0.6338 (3)	−0.03163 (14)	0.0406 (8)	
C6	0.4098 (3)	0.6474 (3)	0.01525 (14)	0.0434 (9)	
C7	0.3344 (3)	0.6321 (3)	0.08455 (16)	0.0555 (11)	
C8	0.3702 (3)	0.7184 (3)	0.08219 (16)	0.0531 (11)	
C9	0.2772 (4)	0.5869 (4)	0.1227 (2)	0.0822 (17)	
H9A	0.2638	0.5254	0.1128	0.123*	
H9B	0.2213	0.6202	0.1267	0.123*	
H9C	0.3099	0.5859	0.1531	0.123*	
C10	0.3611 (5)	0.7958 (5)	0.1173 (2)	0.0863 (19)	
H10A	0.3258	0.7764	0.1450	0.129*	
H10B	0.3309	0.8460	0.1016	0.129*	
H10C	0.4206	0.8147	0.1280	0.129*	
C11	0.6139 (3)	0.7685 (3)	0.09316 (16)	0.0532 (11)	
H11A	0.5641	0.7359	0.1051	0.064*	
C12	0.6937 (3)	0.7703 (3)	0.12029 (17)	0.0557 (11)	
H12A	0.6976	0.7394	0.1499	0.067*	
C13	0.7668 (3)	0.8185 (3)	0.10272 (17)	0.0579 (12)	
H13A	0.8209	0.8210	0.1205	0.070*	
C14	0.7602 (3)	0.8632 (3)	0.05894 (17)	0.0551 (11)	
H14A	0.8098	0.8950	0.0462	0.066*	
C15	0.6777 (3)	0.8597 (2)	0.03390 (15)	0.0412 (8)	
C16	0.6608 (2)	0.9028 (2)	−0.01296 (14)	0.0383 (8)	
C17	0.6694 (3)	0.9807 (3)	−0.08144 (15)	0.0502 (9)	
C18	0.5849 (3)	0.9440 (3)	−0.07755 (15)	0.0479 (10)	
C19	0.7111 (4)	1.0404 (4)	−0.1201 (2)	0.0766 (16)	
H19A	0.6671	1.0515	−0.1454	0.115*	
H19B	0.7636	1.0104	−0.1338	0.115*	
H19C	0.7294	1.0975	−0.1059	0.115*	
C20	0.5027 (4)	0.9551 (4)	−0.1099 (2)	0.0757 (16)	
H20A	0.5189	0.9907	−0.1380	0.114*	
H20B	0.4550	0.9856	−0.0921	0.114*	
H20C	0.4815	0.8961	−0.1201	0.114*	
C21	0.4109 (4)	1.0078 (3)	0.0069 (2)	0.0658 (13)	0.50
H21A	0.4679	1.0357	0.0090	0.079*	0.50
C22	0.2631 (3)	0.9850 (4)	0.0016 (2)	0.075 (5)	0.50
H22A	0.2004	0.9950	−0.0001	0.090*	0.50
N8	0.3313 (4)	1.0509 (4)	0.00487 (19)	0.069 (3)	0.50
H8A	0.3233	1.1091	0.0055	0.083*	0.50
C23	0.3044 (3)	0.9069 (5)	0.0015 (2)	0.0707 (14)	0.50
H23A	0.2751	0.8507	−0.0010	0.085*	0.50
N7	0.3958 (2)	0.9177 (2)	0.00537 (13)	0.0492 (8)	0.50
C23'	0.4109 (4)	1.0078 (3)	0.0069 (2)	0.0658 (13)	0.50
H21B	0.4679	1.0357	0.0090	0.079*	0.50
N8'	0.2631 (3)	0.9850 (4)	0.0016 (2)	0.075 (3)	0.50
H8'A	0.2051	0.9942	0.0000	0.090*	0.50
N7'	0.3958 (2)	0.9177 (2)	0.00537 (13)	0.0492 (8)	0.50
C21'	0.3044 (3)	0.9069 (5)	0.0015 (2)	0.0707 (14)	0.50
H23B	0.2751	0.8507	−0.0010	0.085*	0.50
C22'	0.3313 (4)	1.0509 (4)	0.00487 (19)	0.069 (3)	0.50
H22B	0.3226	1.1138	0.0055	0.083*	0.50
N1	0.4977 (2)	0.7080 (2)	−0.04776 (12)	0.0412 (7)	
N2	0.4179 (2)	0.7263 (2)	0.03865 (12)	0.0463 (8)	
N3	0.3601 (2)	0.5891 (2)	0.04254 (14)	0.0514 (9)	
H3A	0.3468	0.5338	0.0347	0.062*	
N4	0.6058 (2)	0.8120 (2)	0.05027 (12)	0.0420 (7)	
N5	0.5794 (2)	0.8946 (2)	−0.03395 (12)	0.0419 (7)	
N6	0.7161 (2)	0.9535 (2)	−0.04085 (13)	0.0481 (8)	
H6	0.7720	0.9668	−0.0343	0.058*	
Cl1	0.32257 (8)	0.32257 (8)	0.0000	0.0702 (5)	
O11	0.2532 (6)	0.2691 (6)	−0.0184 (2)	0.081 (3)	0.50
O12	0.2849 (7)	0.4105 (5)	0.0068 (4)	0.084 (2)	0.50
O13	0.3603 (6)	0.2882 (6)	0.0403 (3)	0.089 (3)	0.50
O14	0.3877 (6)	0.3325 (6)	−0.0371 (3)	0.080 (2)	0.50
Cl2	0.99578 (7)	0.99578 (7)	0.0000	0.0583 (4)	
O21	0.9987 (3)	0.9145 (2)	0.02515 (14)	0.0775 (11)	
O22	1.0050 (3)	1.0666 (3)	0.03420 (14)	0.0815 (12)	
Cl3	0.66607 (8)	0.15608 (7)	0.02360 (4)	0.0550 (3)	
O31	0.7316 (3)	0.1668 (3)	−0.01093 (13)	0.0798 (12)	
O32	0.6524 (3)	0.2387 (3)	0.04837 (15)	0.0804 (12)	
O33	0.6871 (3)	0.0867 (3)	0.05352 (15)	0.0864 (13)	
O34	0.5931 (3)	0.1353 (3)	−0.00339 (17)	0.0867 (12)	

### Atomic displacement parameters (Å^2^)

**Table:**

	*U*^11^	*U*^22^	*U*^33^	*U*^12^	*U*^13^	*U*^23^
Cu1	0.0378 (2)	0.0426 (3)	0.0483 (2)	−0.00376 (19)	0.0047 (2)	0.0029 (2)
C1	0.043 (2)	0.059 (3)	0.046 (2)	−0.0063 (19)	0.0089 (18)	0.0013 (19)
C2	0.050 (3)	0.074 (3)	0.044 (2)	0.003 (2)	0.0059 (19)	−0.011 (2)
C3	0.069 (3)	0.054 (3)	0.054 (3)	0.003 (2)	−0.006 (2)	−0.015 (2)
C4	0.056 (3)	0.045 (2)	0.063 (3)	−0.0016 (19)	0.001 (2)	−0.010 (2)
C5	0.0354 (19)	0.046 (2)	0.041 (2)	−0.0006 (16)	−0.0021 (16)	−0.0004 (17)
C6	0.041 (2)	0.046 (2)	0.043 (2)	−0.0021 (17)	0.0036 (17)	0.0039 (17)
C7	0.049 (2)	0.063 (3)	0.055 (2)	−0.003 (2)	0.005 (2)	0.011 (2)
C8	0.048 (2)	0.066 (3)	0.046 (2)	−0.006 (2)	0.0104 (19)	0.002 (2)
C9	0.084 (4)	0.086 (4)	0.077 (4)	−0.002 (3)	0.033 (3)	0.025 (3)
C10	0.099 (5)	0.092 (4)	0.068 (3)	−0.016 (4)	0.025 (3)	−0.022 (3)
C11	0.059 (3)	0.047 (2)	0.054 (3)	−0.007 (2)	−0.007 (2)	0.014 (2)
C12	0.061 (3)	0.049 (2)	0.057 (3)	−0.001 (2)	−0.015 (2)	0.006 (2)
C13	0.061 (3)	0.054 (3)	0.059 (3)	0.007 (2)	−0.023 (2)	−0.004 (2)
C14	0.043 (2)	0.057 (3)	0.066 (3)	−0.0044 (19)	−0.005 (2)	−0.006 (2)
C15	0.040 (2)	0.0344 (18)	0.049 (2)	−0.0007 (16)	−0.0077 (17)	−0.0044 (16)
C16	0.0306 (18)	0.0389 (19)	0.045 (2)	−0.0029 (15)	0.0010 (15)	0.0024 (16)
C17	0.055 (2)	0.046 (2)	0.050 (2)	0.0019 (19)	0.0063 (19)	0.0026 (18)
C18	0.047 (2)	0.049 (2)	0.048 (2)	0.0022 (18)	0.0001 (18)	0.0077 (18)
C19	0.065 (3)	0.079 (4)	0.086 (4)	−0.009 (3)	0.021 (3)	0.025 (3)
C20	0.072 (3)	0.095 (4)	0.060 (3)	0.003 (3)	−0.010 (3)	0.025 (3)
C21	0.083 (4)	0.044 (2)	0.071 (3)	−0.003 (2)	0.005 (3)	0.005 (2)
C22	0.055 (9)	0.089 (11)	0.081 (8)	0.02 (11)	0.01 (11)	0.02 (13)
N8	0.070 (7)	0.068 (7)	0.070 (6)	0.02 (10)	0.01 (9)	0.02 (9)
C23	0.046 (3)	0.095 (4)	0.071 (3)	−0.006 (3)	0.008 (3)	−0.011 (3)
N7	0.0397 (18)	0.0479 (19)	0.060 (2)	0.0012 (14)	−0.0002 (17)	−0.0015 (17)
C23'	0.083 (4)	0.044 (2)	0.071 (3)	−0.003 (2)	0.005 (3)	0.005 (2)
N8'	0.055 (6)	0.089 (6)	0.081 (3)	0.02 (10)	0.01 (9)	0.02 (11)
N7'	0.0397 (18)	0.0479 (19)	0.060 (2)	0.0012 (14)	−0.0002 (17)	−0.0015 (17)
C21'	0.046 (3)	0.095 (4)	0.071 (3)	−0.006 (3)	0.008 (3)	−0.011 (3)
C22'	0.070 (6)	0.068 (6)	0.070 (3)	0.02 (11)	0.01 (11)	0.02 (10)
N1	0.0327 (16)	0.0462 (18)	0.0449 (17)	−0.0019 (14)	0.0006 (14)	−0.0035 (14)
N2	0.0441 (19)	0.051 (2)	0.0436 (17)	−0.0092 (15)	0.0047 (15)	−0.0012 (16)
N3	0.050 (2)	0.0419 (18)	0.062 (2)	−0.0091 (15)	0.0039 (17)	0.0088 (17)
N4	0.0422 (17)	0.0372 (17)	0.0465 (18)	−0.0048 (14)	−0.0034 (14)	0.0044 (14)
N5	0.0346 (16)	0.0450 (18)	0.0460 (17)	−0.0004 (13)	0.0029 (14)	0.0051 (14)
N6	0.0363 (17)	0.049 (2)	0.059 (2)	−0.0059 (14)	−0.0003 (16)	0.0036 (16)
Cl1	0.0593 (6)	0.0593 (6)	0.0920 (13)	−0.0108 (8)	−0.0109 (8)	0.0109 (8)
O11	0.078 (6)	0.075 (5)	0.090 (6)	−0.035 (4)	0.034 (5)	−0.009 (5)
O12	0.102 (6)	0.052 (4)	0.098 (6)	−0.019 (4)	0.015 (6)	−0.027 (5)
O13	0.099 (6)	0.096 (6)	0.072 (5)	−0.050 (5)	−0.016 (5)	0.027 (4)
O14	0.095 (6)	0.074 (5)	0.072 (5)	−0.019 (5)	0.031 (5)	−0.014 (4)
Cl2	0.0531 (5)	0.0531 (5)	0.0686 (9)	−0.0092 (6)	0.0084 (6)	−0.0084 (6)
O21	0.094 (3)	0.0521 (19)	0.087 (2)	−0.0083 (19)	−0.043 (2)	0.0116 (18)
O22	0.093 (3)	0.074 (2)	0.078 (2)	−0.033 (2)	0.039 (2)	−0.0269 (19)
Cl3	0.0676 (7)	0.0422 (5)	0.0552 (6)	−0.0112 (5)	−0.0081 (5)	−0.0014 (4)
O31	0.072 (2)	0.097 (3)	0.070 (2)	−0.031 (2)	0.0292 (18)	−0.037 (2)
O32	0.078 (2)	0.064 (2)	0.099 (3)	0.0249 (19)	−0.039 (2)	−0.028 (2)
O33	0.085 (3)	0.083 (3)	0.092 (3)	0.039 (2)	−0.035 (2)	0.013 (2)
O34	0.095 (3)	0.065 (2)	0.101 (3)	−0.016 (2)	−0.024 (2)	−0.024 (2)

### Geometric parameters (Å, °)

**Table:**

Cu1—N5	1.977 (3)	C18—N5	1.398 (5)
Cu1—N2	1.990 (3)	C18—C20	1.503 (7)
Cu1—N7	2.007 (3)	C19—H19A	0.9600
Cu1—N1	2.129 (3)	C19—H19B	0.9600
Cu1—N4	2.137 (3)	C19—H19C	0.9600
C1—N1	1.327 (5)	C20—H20A	0.9600
C1—C2	1.380 (6)	C20—H20B	0.9600
C1—H1A	0.9300	C20—H20C	0.9600
C2—C3	1.380 (7)	C21—N8	1.326 (7)
C2—H2A	0.9300	C21—N7	1.338 (6)
C3—C4	1.383 (7)	C21—H21A	0.9300
C3—H3B	0.9300	C22—C23	1.293 (8)
C4—C5	1.407 (6)	C22—N8	1.392 (8)
C4—H4A	0.9300	C22—H22A	0.9300
C5—N1	1.352 (5)	N8—H8A	0.8600
C5—C6	1.437 (5)	C23—N7	1.352 (6)
C6—N2	1.326 (5)	C23—H23A	0.9300
C6—N3	1.347 (5)	N3—H3A	0.8600
C7—N3	1.364 (6)	N6—H6	0.8600
C7—C8	1.369 (6)	Cl1—O13	1.334 (8)
C7—C9	1.493 (6)	Cl1—O13^i^	1.334 (8)
C8—N2	1.386 (5)	Cl1—O11^i^	1.378 (7)
C8—C10	1.492 (7)	Cl1—O11	1.378 (7)
C9—H9A	0.9600	Cl1—O14^i^	1.401 (8)
C9—H9B	0.9600	Cl1—O14	1.401 (8)
C9—H9C	0.9600	Cl1—O12	1.413 (8)
C10—H10A	0.9600	Cl1—O12^i^	1.413 (8)
C10—H10B	0.9600	O11—O11^i^	1.060 (15)
C10—H10C	0.9600	O11—O13^i^	1.551 (10)
C11—N4	1.342 (5)	O12—O14^i^	1.135 (11)
C11—C12	1.385 (6)	O12—O13^i^	1.486 (11)
C11—H11A	0.9300	O13—O12^i^	1.486 (11)
C12—C13	1.369 (7)	O13—O14^i^	1.515 (13)
C12—H12A	0.9300	O13—O11^i^	1.551 (10)
C13—C14	1.369 (6)	O14—O12^i^	1.135 (11)
C13—H13A	0.9300	O14—O13^i^	1.515 (13)
C14—C15	1.391 (6)	Cl2—O21^i^	1.375 (3)
C14—H14A	0.9300	Cl2—O21	1.375 (3)
C15—N4	1.340 (5)	Cl2—O22	1.403 (3)
C15—C16	1.452 (5)	Cl2—O22^i^	1.403 (3)
C16—N5	1.327 (5)	Cl3—O34	1.334 (4)
C16—N6	1.338 (5)	Cl3—O33	1.341 (4)
C17—C18	1.353 (6)	Cl3—O31	1.356 (3)
C17—N6	1.365 (5)	Cl3—O32	1.402 (4)
C17—C19	1.504 (6)		
			
N5—Cu1—N2	170.20 (15)	C23—C22—N8	106.1 (4)
N5—Cu1—N7	94.06 (14)	C23—C22—H22A	127.0
N2—Cu1—N7	95.74 (15)	N8—C22—H22A	127.0
N5—Cu1—N1	94.06 (13)	C21—N8—C22	107.7 (5)
N2—Cu1—N1	80.10 (13)	C21—N8—H8A	126.2
N7—Cu1—N1	127.46 (14)	C22—N8—H8A	126.2
N5—Cu1—N4	79.52 (13)	C22—C23—N7	111.1 (5)
N2—Cu1—N4	94.82 (13)	C22—C23—H23A	124.4
N7—Cu1—N4	123.73 (14)	N7—C23—H23A	124.4
N1—Cu1—N4	108.79 (13)	C21—N7—C23	106.3 (4)
N1—C1—C2	122.9 (4)	C21—N7—Cu1	126.7 (3)
N1—C1—H1A	118.6	C23—N7—Cu1	126.7 (4)
C2—C1—H1A	118.6	C1—N1—C5	118.3 (3)
C1—C2—C3	119.3 (4)	C1—N1—Cu1	129.5 (3)
C1—C2—H2A	120.4	C5—N1—Cu1	112.2 (2)
C3—C2—H2A	120.4	C6—N2—C8	107.4 (3)
C2—C3—C4	119.4 (4)	C6—N2—Cu1	113.6 (3)
C2—C3—H3B	120.3	C8—N2—Cu1	139.0 (3)
C4—C3—H3B	120.3	C6—N3—C7	109.0 (4)
C3—C4—C5	117.8 (4)	C6—N3—H3A	125.5
C3—C4—H4A	121.1	C7—N3—H3A	125.5
C5—C4—H4A	121.1	C15—N4—C11	118.1 (4)
N1—C5—C4	122.4 (4)	C15—N4—Cu1	112.9 (3)
N1—C5—C6	113.2 (3)	C11—N4—Cu1	129.0 (3)
C4—C5—C6	124.4 (4)	C16—N5—C18	105.8 (3)
N2—C6—N3	109.3 (4)	C16—N5—Cu1	114.9 (3)
N2—C6—C5	120.9 (4)	C18—N5—Cu1	139.2 (3)
N3—C6—C5	129.7 (4)	C16—N6—C17	108.8 (3)
N3—C7—C8	106.2 (4)	C16—N6—H6	125.6
N3—C7—C9	122.7 (4)	C17—N6—H6	125.6
C8—C7—C9	131.0 (5)	O13—Cl1—O13^i^	177.0 (6)
C7—C8—N2	108.1 (4)	O13—Cl1—O11^i^	69.8 (5)
C7—C8—C10	129.4 (4)	O13^i^—Cl1—O11^i^	113.2 (5)
N2—C8—C10	122.5 (4)	O13—Cl1—O11	113.2 (5)
C7—C9—H9A	109.5	O13^i^—Cl1—O11	69.8 (5)
C7—C9—H9B	109.5	O11^i^—Cl1—O11	45.3 (6)
H9A—C9—H9B	109.5	O13—Cl1—O14^i^	67.2 (6)
C7—C9—H9C	109.5	O13^i^—Cl1—O14^i^	111.0 (6)
H9A—C9—H9C	109.5	O11^i^—Cl1—O14^i^	107.1 (5)
H9B—C9—H9C	109.5	O11—Cl1—O14^i^	136.7 (5)
C8—C10—H10A	109.5	O13—Cl1—O14	111.0 (6)
C8—C10—H10B	109.5	O13^i^—Cl1—O14	67.2 (6)
H10A—C10—H10B	109.5	O11^i^—Cl1—O14	136.7 (5)
C8—C10—H10C	109.5	O11—Cl1—O14	107.1 (5)
H10A—C10—H10C	109.5	O14^i^—Cl1—O14	112.7 (7)
H10B—C10—H10C	109.5	O13—Cl1—O12	113.4 (6)
N4—C11—C12	122.3 (4)	O13^i^—Cl1—O12	65.4 (6)
N4—C11—H11A	118.8	O11^i^—Cl1—O12	113.7 (6)
C12—C11—H11A	118.8	O11—Cl1—O12	106.2 (6)
C13—C12—C11	118.7 (4)	O14^i^—Cl1—O12	47.6 (5)
C13—C12—H12A	120.6	O14—Cl1—O12	105.5 (5)
C11—C12—H12A	120.6	O13—Cl1—O12^i^	65.4 (6)
C12—C13—C14	119.9 (4)	O13^i^—Cl1—O12^i^	113.4 (6)
C12—C13—H13A	120.0	O11^i^—Cl1—O12^i^	106.2 (6)
C14—C13—H13A	120.0	O11—Cl1—O12^i^	113.7 (6)
C13—C14—C15	118.4 (4)	O14^i^—Cl1—O12^i^	105.5 (5)
C13—C14—H14A	120.8	O14—Cl1—O12^i^	47.6 (5)
C15—C14—H14A	120.8	O12—Cl1—O12^i^	136.7 (7)
N4—C15—C14	122.4 (4)	O11^i^—O11—Cl1	67.4 (3)
N4—C15—C16	112.9 (3)	O11^i^—O11—O13^i^	119.0 (5)
C14—C15—C16	124.6 (4)	Cl1—O11—O13^i^	53.8 (4)
N5—C16—N6	110.2 (3)	O14^i^—O12—Cl1	65.7 (6)
N5—C16—C15	119.8 (3)	O14^i^—O12—O13^i^	118.1 (9)
N6—C16—C15	130.0 (4)	Cl1—O12—O13^i^	54.7 (4)
C18—C17—N6	106.2 (4)	Cl1—O13—O12^i^	59.8 (5)
C18—C17—C19	131.2 (4)	Cl1—O13—O14^i^	58.5 (5)
N6—C17—C19	122.6 (4)	O12^i^—O13—O14^i^	96.5 (6)
C17—C18—N5	108.9 (4)	Cl1—O13—O11^i^	56.4 (4)
C17—C18—C20	130.0 (4)	O12^i^—O13—O11^i^	94.5 (6)
N5—C18—C20	120.9 (4)	O14^i^—O13—O11^i^	93.6 (7)
C17—C19—H19A	109.5	O12^i^—O14—Cl1	66.7 (6)
C17—C19—H19B	109.5	O12^i^—O14—O13^i^	119.4 (8)
H19A—C19—H19B	109.5	Cl1—O14—O13^i^	54.3 (4)
C17—C19—H19C	109.5	O21^i^—Cl2—O21	107.7 (3)
H19A—C19—H19C	109.5	O21^i^—Cl2—O22	113.3 (3)
H19B—C19—H19C	109.5	O21—Cl2—O22	107.5 (2)
C18—C20—H20A	109.5	O21^i^—Cl2—O22^i^	107.5 (2)
C18—C20—H20B	109.5	O21—Cl2—O22^i^	113.3 (3)
H20A—C20—H20B	109.5	O22—Cl2—O22^i^	107.7 (3)
C18—C20—H20C	109.5	O34—Cl3—O33	110.5 (3)
H20A—C20—H20C	109.5	O34—Cl3—O31	101.9 (3)
H20B—C20—H20C	109.5	O33—Cl3—O31	110.6 (3)
N8—C21—N7	108.8 (5)	O34—Cl3—O32	110.5 (2)
N8—C21—H21A	125.6	O33—Cl3—O32	113.0 (3)
N7—C21—H21A	125.6	O31—Cl3—O32	109.8 (3)

Symmetry codes: (i) *y*, *x*, −*z*.

### Hydrogen-bond geometry (Å, °)

**Table:**

*D*—H···*A*	*D*—H	H···*A*	*D*···*A*	*D*—H···*A*
N3—H3A···O12	0.86	2.16	3.001 (8)	166
N3—H3A···O14^i^	0.86	2.15	2.980 (10)	162
N6—H6···O21^i^	0.86	2.15	3.009 (5)	175

Symmetry codes: (i) *y*, *x*, −*z*.
